# Letter from F. K. Bailey, M.D.

**Published:** 1871-03

**Authors:** F. K. Bailey


### Correspondence.

---

PROF. DAVIS,—DEAR SIR,—Knoxville and vicinity has been very free from severe disease during the last ten months, and surgical cases comparatively rare.

Fevers occasionally occur, of a simple ephemeral character, attended with violent headache, pain in the back and limbs, with nausea. There is generally constipation, very red, scanty urine, and a hot, dry skin, tongue loaded with a white fur. The administration of mercurials, followed by saline cathartics, generally control the disease in less than five days.

Antiperiodics are seldom called for, and cinchona and its preparations are merely required as general tonics. A case of simple intermittent is never seen, unless imported from the South or West.

I have had during the last four weeks a case of continued fever, in which there was a stage of incubation lasting nearly a week. When I was called, the patient, a white boy four years old, complained of a pain in the head and stomach, and loss of appetite. There was moderate looseness of the bowels, but no diarrhœa; sweating at night during sleep, but no coldness of the extremities. There was also slight wildness on first awaking, and disposition to cry out from fear.

With all the above morbid appearances, the tongue was nearly natural in appearance, having no coat, and but slightly reddened. The papillæ were not at all elevated, nor could any sordes be discovered in or about the mouth.

There were exacerbations noticeable on each alternate night, but not severe, and, with that exception, the febrile reaction continued very uniform throughout, until the fourteenth day, when the little fellow commenced improving rapidly. On the sixteenth day he was dressed and walking about the house. About the tenth day there was slight increased action of the bowels, but it was checked by a single dose of subnitrate of bismuth. The treatment consisted in small doses of Dover's powder, and ½ gr. sul. quin. every four hours, alternated with a teaspoonful of the following mixture:

℞ Brom. Potassii,------------------------℈j.

Spts. Ether Comp.,--------------------ʒi.

Tr. Sang. Canad,------------------ʒij.

Tr. Cinchonæ,--------------------------ʒv.

Syrupi Simp.,--------------------------℥j.

Diet of beef-tea and milk.

Such cases are comparatively so rare here that I have considered this exceptional.

In observing the effects of bromide of potassium, I have noticed two cases in which the pulse went down to 50 in a minute under its use. One was that of a young man, who, from overwork as salesman in a wholesale store, induced a fever, attended with hepatic engorgement, and slight congestion in the right lung.

After febrile excitement had subsided, insomnolence intervened, with slight delirium towards morning. To obviate this, I ordered bromide potassii, in 15 gr. doses, every four hours, commencing at 6 P.M. Instead of inducing sleep, the pulse lessened, with an aggravation of the hallucination. A few doses of spts. ether comp. brought up the pulse and quieted excitement.

The other case was that of a young man belonging to a minstrel troupe travelling with Col. Ames' circus. He had been exposed to wet and fatigue in crossing the hilly regions from Kentucky to East Tennessee.

Extremely nervous temperament, besides a sufferer from tertiary syphilis, as shown by cicatrices in the cervical region, and his history, as given by himself.

He was complaining when I saw him first, of lancinating pains through both knees, and extending downward in front part of the legs. Full doses of morphine failing to relieve the the first night, I gave him 15 or 20 grs. bromide potassii for three or four times, when he became frantic, and I was called about midnight. Found his pulse less than 50, and the pain increased. Gave Hoffman's anodyne with brandy, and commenced quinine in 5 grain doses, to be given during the day with iod. potassii, 8 grs.

There was no return of the periosteal pain, and he was soon able to be about.

In asthenic cases, where the bromide is used, I combine ether, camphor, or other diffusable stimulant.

January 20th I was called to treat a colored child 25 days old, laboring under epileptic convulsions. After giving small doses of calomel to act upon the bowels, commenced with bromide potassii and bromide ammonia, each one grain every four hours, alternated with quinine and pulv. Dover.

In a day or two the fits stopped, and did not return for more than a week. I then commenced giving 2 grs. chloral every four hours in syrup, which almost immediately stopped a recurrence of the convulsions. For two days there have been but slight indications of a return.

Any agent which will control the epilepsy of new born infants, so common among the blacks, will prove a boon.

The weather during the holidays was unusually cold, as the mercury sank to zero, which has not occurrred more than four times before for fifty years.

Snow fell at one time to the depth of three inches, and ice formed six inches in thickness.

Since the 1st instant it has not been cold enough to freeze water, and, when the sun is not obscured by clouds, it is warm and springlike. A few cases of scarlatina have occurred, but of a mild type, and no deaths.

In families where children have been affected, adults have been attacked with a sore throat, and febrile reaction would be severe for some days.

Suppurative tonsilitis has been quite prevalent, and an unusual number of people are suffering from boils about the neck.

The anginose symptoms are seldom seen in children, but the eruption very fully developed. The desquamation is delayed somewhat, and in some cases hardly occurs. No treatment but good care and palliation is required.

The East Tennessee Medical Society was reorganized, or rather renewed on the 2d instant.

There are about twenty regular physicians in Knoxville, most of them will become active members. The last meeting was held in October, 1860. The Society will hold monthly meetings for the discussion of medical subjects, and the reporting of cases. Embracing a section of country composed of thirty counties it should be a channel of communication for a great number of medical men.

In closing this communication I desire to say a few words in regard to this section of country as a health resort. Much inquiry of late is made upon this subject, and many persons come every autumn to Knoxville and other points in this valley to spend the winter. To sufferers from asthma it may be said that no doubt exists as to the advantages possessed here. People from Maine to Ohio have been materially benefitted by a residence of one or two years.

A gentleman from Maine came here two years ago so much affected with asthma that he could not climb a flight of stairs without causing distressing dyspnœa, who now is engaged in active mercantile business, and feels very much better than when he came.

A lady who came from Ohio in 1866, has been so much relieved from asthma, which commenced in childhood, that she does not have a paroxysm once in six months.

On returning to Ohio last fall to visit her friends, she states that the symptoms began to be felt as soon as she reached her old home, and that before she had been at her father's ten days the demon returned with all its former violence. On returning she declared she would never go back again, for the annoyance was too great to suffer while she ought to be enjoying the society of friends.

To sufferers from malarial diseases, in whatever region of our common country they may reside, it may be said with certainty, that this equable mountian air will prove a cure. Of this I speak with assurance, from its influence upon my own health, and others can assert the same.

FEBRUARY, 1871. F. K. BAILEY, M.D.

